# The impact of *IDH* and *NAT2* gene polymorphisms in acute myeloid leukemia risk and overall survival in an Arab population: A case-control study

**DOI:** 10.1371/journal.pone.0289014

**Published:** 2023-07-21

**Authors:** Sohaib M. Al-Khatib, Obada Ababneh, Hassann Abushukair, Nour Abdo, Laith N. Al-Eitan

**Affiliations:** 1 Department of Pathology and Laboratory Medicine, Faculty of Medicine, Jordan University of Science and Technology, Irbid, Jordan; 2 Faculty of Medicine, Jordan University of Science and Technology, Irbid, Jordan; 3 Department of Public Health, Faculty of Medicine, Jordan University of Science and Technology, Irbid, Jordan; 4 Department of Biotechnology and Genetic Engineering, Faculty of Science and Arts, Jordan University of Science and Technology, Irbid, Jordan; CNR, ITALY

## Abstract

Acute myeloid leukemia (AML) is a malignancy of the myeloid cells due to the clonal and malignant proliferation of blast cells. The etiology of AML is complex and involves environmental and genetic factors. Such genetic aberrations include *FLT3*, *DNMT3*, *IDH1*, *IDH2*, *NAT2*, *and WT*. In this study, we analyzed the relationship between five, not previously studied in any Arab population, single nucleotide polymorphisms (SNPs) and the risk and overall survival of AML in Jordanian patients. The SNPs are *NAT2* (rs1799930 and rs1799931), *IDH1* (rs121913500), and *IDH2* (rs121913502 and rs1057519736). A total number of 30 AML patients and 225 healthy controls were included in this study. Females comprised 50% (n = 15) and 65.3% (n = 147) of patients and controls, respectively. For AML patients (case group) Genomic DNA was extracted from formalin-fixed paraffin-embedded tissues and from peripheral blood samples for the control subjects group. Genotyping of the genetic polymorphisms was conducted using a sequencing protocol. Our study indicates that *NAT2* rs1799930 SNP had a statistically significant difference in genotype frequency between cases and controls (p = 0.023) while *IDH* mutations did not correlate with the risk and survival of AML in the Jordanian population. These results were also similar in the TCGA-LAML cohorts with the notable exception of the rare *NAT2* mutation. A larger cohort study is needed to further investigate our results.

## Introduction

Acute myeloid leukemia (AML) is a malignancy of the myeloid cells due to the clonal and malignant proliferation of blast cells [1]. AML is the most prevalent type of acute leukemia in adults with a median age of 68 years. According to Globocan report, the 5-year prevalence of leukemia is 15.26 per 100000 in Jordan in 2020 [2]. The etiology of AML is complex and involves environmental and genetic factors [3]. Such environmental factors include radiation, smoking, obesity, and previous exposure to chemotherapy or radiation; and genetic aberrations include *FLT3*, *DNMT3*, *IDH1*, *IDH2*, *NAT2*, *and WT1* [4, 5]. As a result, research into somatic mutations and single-nucleotide polymorphisms (SNPs) has aided in gaining a better understanding of the mechanisms driving leukemogenesis, treatment response and improving patients’ survival [6].

N-acetyltransferase 2 *(NAT2)* is a gene located in the short arm of chromosome 8 and plays a key role in metabolizing carcinogens such as aromatic amines, arylamines and hydrazines throughout acetylation reactions [7]. Such compounds are also found in cigarette smoke [8]. Different SNPs of *NAT-2* have been identified which mark up to distinct populations, slow and rapid acetylator [9]. NAT*4 is the wild type form and considered the main example of rapid acetylator while the absence of it with the presence of the other alleles like rs1799930 and rs1799931 denotes a slow acetylator [10]. These variations have been discussed in light of autoimmunity, tuberculosis treatment, Parkinson’s disease, and cancer [11–13]. Previous studies have shown a link between *NAT2* slow acetylation phenotype and bladder, lung, colon, breast, liver, and gastric cancers [14–17]. Although some studies showed an association of *NAT2* with increased risk and drug response in terms of AML, the results are conflicting with some studies showing these phenotypes did not affect AML risk [18–20].

Isocitrate dehydrogenases (*IDH 1 and IDH2*) are part of the tricarboxylic cycle and catalyze the reversible reaction between iso-citrate and α-ketoglutarate. *IDH* mutations have been heavily studied in brain gliomas [21]. It has been found that *IDH* mutations are closely related to the occurrence and prognosis of glioma [21]. Recently, the role of *IDH* mutations has been also investigated in AML. The incidence of *IDH1* and *IDH2* mutations have been associated with leukemogenesis with an incidence ranging from 8% to 12% [22]. These mutations have been associated with leukemogenesis by preventing the revisable reaction of isocitrate to alpha-ketoglutarate in the tricarboxylic acid cycle and, instead, lead to increased production of the oncogenic 2-hydroxyglutarate from alpha-ketoglutarate [23]. However, there is a conflicting evidence about *IDH1/2* mutations’ predictive effect on AML [24].

The aim of this study was, therefore, to analyze the relationship between five, not previously studied in any Arab population, single nucleotide polymorphisms (SNPs) and the risk and overall survival of AML in Jordanian Arab patients. The SNPs are *NAT2* (rs1799930 and rs1799931), *IDH1* (rs121913500), and *IDH2* (rs121913502 and rs1057519736). In addition, we wanted to explore the value of these genes in publicly curated data at the multi-omics level.

## Methods and materials

### Patients and data collection

Paraffin-embedded samples from AML patients (n = 30) were retrieved from the archives of King Abdullah University Hospital during the period of January 2013 to December 2021. All cases were reviewed by (SK) and one representative section was chosen from each case. The human ethics approval was attained by the ethical committee of Jordan University of Science and Technology [Institutional Review Board (IRB) code number 18/105/2017, dated 04/05/2017] in accordance with the 1964 Declaration of Helsinki and its later amendments. Formal written informed consent was not required with a waiver by the IRB. Control group samples were peripheral blood and all healthy controls (n = 225) were voluntarily involved and signed written informed consent. Cases’ and controls’ names were coded and blinded and treated confidentially. Authors had no access to information that could identify individual participants during or after data collection.

### DNA extraction

Genomic DNA was extracted for the AML patients from formalin-fixed paraffin-embedded tissue using a commercially available kit, DNeasy Blood & Tissue Kit (Qiagen Ltd., West Sussex, UK), using the manufacturer’s protocols. Genomic DNA from control-subjects’ blood samples was extracted using the QIAamp® or Promega DNA Mini Kit according to the manufacturer’s instruction. The quality of extracted DNA was examined using agarose gel electrophoresis and ethidium bromide staining. The concentration and purity of extracted DNA were assessed using a NanoDrop 1000® spectrophotometer. The pure DNA samples with their concentrations were sent to the Australian Genome Research Facility (AGRF, Melbourne Node, Melbourne, Australia) for genotyping of five SNPs *NAT2* (rs1799930 and rs1799931), *IDH1* (rs121913500), and *IDH2* (rs121913502 and rs1057519736) in all subjects (patients and controls). The SNPs, SNPs’ position, and primer sequences are shown in **Table 1**. Genotyping with the Sequenom MassARRAY® system (iPLEX GOLD) (Sequenom, San Diego, CA, USA) was performed at the AGRF according to the manufacturer’s recommendations (Sequenom, San Diego, CA, USA). Genotype distributions were compared between patients and controls. Unconditional logistic regression analysis was used to estimate the association between the genotype frequency and the risk of developing AML.

**Table 1:** The SNPs, SNPs positions and primers sequences for *NAT2*, *IDH1*, and IDH2.

SNP-ID	Gene	Chr^	PCR Primer 1	PCR Primer 2
**rs1799930**	*NAT2*	8p22	ACGTTGGATGAAGATGTTGGAGACGTCTGC	ACGTTGGATGCCTGCCAAAGAAGAAACACC
**rs1799931**	*NAT2*	8p22	ACGTTGGATGGGAAGAGGTTGAAGAAGTGC	ACGTTGGATGGGGTGATACATACACAAGGG
**rs121913500**	*IDH1*	2q34	ACGTTGGATGACATGACTTACTTGATCCCC	ACGTTGGATGAAAATATCCCCCGGCTTGTG
**rs121913502**	*IDH2*	15q26.1	ACGTTGGATGCTAGGCGTGGGATGTTTTTG	ACGTTGGATGATGCTAGTCAGGTAGTGCTC
**rs1057519736**	*IDH2*	15q26.1	ACGTTGGATGAGGTCAGTGGATCCCCTCTC	ACGTTGGATGAAAACATCCCACGCCTAGTC

^ Chr: Chromosome.

### TCGA analysis

To further understand the impact of included genes on AML patients, the cancer genomic atlas (TCGA) acute myeloid leukemia pancancer atlas cohort was included [25]. Mutational and RNAseq data were obtained to investigate the prognostic value of our genes of interest in terms of expression and mutational status. Data on 200 AML patients were extracted from the TCGA project through cBioPortal and survival analysis as conducted using the web-based computation tool UCSC Xena tool [26, 27]. Oncoprint graphs were generated using cBioPortal to illustrate the genomics features of the included cohort. Cut-off points for mRNA expression data was generated using the X-Tile program [28].

### Statistical analysis

Categorical variables were reported as the number of cases (percentage) and compared using the Pearson’s Chi square (χ2) test or Fisher’s exact test as appropriate. Continuous variables were expressed as mean ± standard deviation (SD) if normally distributed and compared using the independent Student’s t test or one-way analysis of variance (ANOVA) as appropriate. The probabilities of Event-free survival (EFS) and Overall survival (OS) were estimated using the Kaplan–Meier method and were compared among subsets of patients using the log-rank test. For all statistical analyses, the P values were two-sided, and a P value of <0.05 was deemed statistically significant. Data statistical analyses were performed using the statistical package for the social sciences (SPSS Statistics for Windows, Version 20.0; IBM Corp., Armonk, NY, USA). Genotypes and alleles frequency was estimated. Genotype frequencies were compared with the frequencies expected by the Hardy–Weinberg equilibrium (HWE) using a χ2 goodness of fit test.

## Results

### Patients characteristic

A total number of 30 AML patients and 225 healthy controls were included in this study. Females comprised 50% (n = 15) and 65.3% (n = 147) of patients and controls, respectively. As for age, patients had a significantly higher mean age compared to controls (48.3 vs 30.6). Upon retrieval of patient’s data, 19 (63.3%) of AML patients were dead and median overall survival was 11 months. Mean Serum LDH for our patients’ cohort was 1013.7 with a substantial standard deviation (SD) of 1045.4. Additional clinical and demographic data are shown in **Table 2**.

**Table 2:** Demographic and clinical data of AML patients and healthy controls of Jordanian Arab descent included in this study.

Demographic Data	Cases (n = 30)	Controls (n = 225)
**Gender (%)**		
Male	15 (50)	78 (34.7)
Female	15 (50)	147 (65.3)
**Age (%)**		
0–14	1 (3.3)	5 (2.2)
15–19	1 (3.3)	27 (12)
20–40	8 (26.7)	149 (66.2)
41–55	7 (23.3)	36 (16)
>55	13 (43.3)	8 (3.6)
Mean (SD)	48.3 (18.3)	30.6 (11.8)
**Clinical Data**		
**Survival Status**		-
Alive	11 (36.7)	-
Dead	19 (63.3)	-
**Overall Survival (Months)**		-
Median	11	-
**Serum LDH**		-
Mean (SD)	1013.7 (1045.4)	-
**Total Protein**		-
Mean (SD)	67.7 (14.9)	-
**Serum Albumin**		-
Mean (Range)	35.9 (8.8)	-
**Platelets**		**-**
Mean (Range)	96.4 (122.8)	-
**Hgb**		-
Mean (Range)	8.6 (2.6)	-
**WBC**		-
Mean (Range)	37.7	-
**Total Monocyte**		-
Mean (Range)	19.1	-

### Allele frequency

For our 5 SNP panel, 4 SNPs had no significant differences between cases and controls when comparing single allele as well as genotype frequency as demonstrated in **Table 3**. The *NAT2* rs1799930 SNP had a statistically significant difference in genotype frequency between cases and controls as the GG genotype was the most common among the cases (69%), whereas the GA genotype was the most common among controls (47%). Allele frequency as 79% for G in patients and 67% for controls.

**Table 3:** Allele frequency and percentage across AML patients and controls.

SNP ID			
rs1799930	Cases N (%)	Controls N (%)	*p*-value
Allele G	46(79)	292(67)	0.058
Allele A	12(21)	144(33)	
NA	2	14	
Genotype A/A	3(10)	21(10)	**0.023**
Genotype G/A	6(21)	102(47)	
Genotype G/G	20(69)	95(44)	
NA	1	7	
**rs1799931**			
Allele G	53(95)	383(98)	0.119**
Allele A	3(5)	7(2)	
NA	4	60	
Genotype G/A	3(11)	7(4)	0.116
Genotype G/G	25(89)	188(96)	
NA	2	30	
**rs121913500**			
Allele C	60(100)	450(100)	----
**rs121913502**			
Allele C	59(98)	450(100)	0.118**
Allele T	1(2)	0	
Genotype C/C	29(97)	225(100)	0.105**
Genotype C/T	1(3)	0	
**rs1057519736**			
Allele G	60(100)	444(100)	----
NA	0	6	

** P-value retrieved from Fisher’s Exact Test in case cell count is less than 5 where Chi-square is not valid

### Genetic models comparison

Four modes of inheritance were considered for our SNPs of interest; codominant, dominant, recessive and overdominant. For the rs1799930 SNP, significant differences were found in the codominant model in which the GA genotype was more frequent among controls (OR: 3.58, 95% CI: 1.38–9.29). The dominant model as well showed a significant difference in which the GG genotype had more odds among cases (OR: 2.88, 95% CI: 1.25–6.61). Lastly, the overdominant model also showed a significant difference in which GG and AA genotypes were more common in the cases cohort (OR: 3.37, 95% CI: 1.32–8.60) **Table 3**.

**Table 4** includes details on the rs1799930 modes of inheritance.

**Table 4:** Stratified analysis based on mode of inheritance for *NAT2* SNP (rs1799930) across AML patients and healthy controls.

Model	Genotype	Case n (%)	Control n (%)	OR (95% CI)	P-value	AIC	BIC
**Codominant**	G/G	20 (69)	95 (43.6)	1	0.018	176.7	187.2
	G/A	6 (20.7)	102 (46.8)	3.58 (1.38–9.29)			
	A/A	3 (10.3)	21 (9.6)	1.47 (0.40–5.42)			
**Dominant**	G/G	20 (69)	95 (43.6)	1	0.0096	176	183
	G/A-A/A	9 (31)	123 (56.4)	2.88 (1.25–6.61)			
**Recessive**	G/G-G/A	26 (89.7)	197 (90.4)	1	0.9	182.7	189.7
	A/A	3 (10.3)	21 (9.6)	0.92 (0.26–3.31)			
**Overdominant**	G/G-A/A	23 (79.3)	116 (53.2)	1	0.0057	175.1	182.1
	G/A	6 (20.7)	102 (46.8)	3.37 (1.32–8.60)			

OR: Odds ratio

AIC: Akaike information criterion

BIC: Bayesian information criterion

### Survival analysis

**Fig 1:**
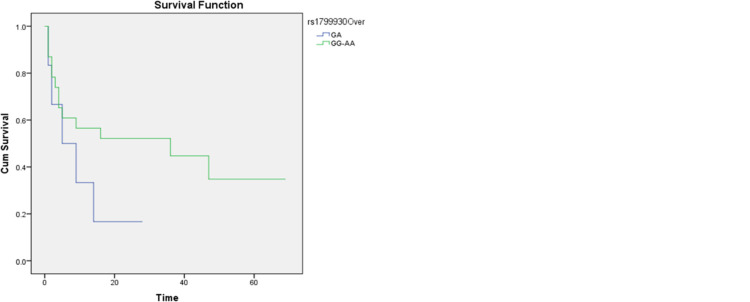
Kaplan Meier plots demonstrating the probability of overall survival based on the *NAT2* rs1799930 SNP according to different modes of inheritance, (a) dominant and (b) overdominant.

Kaplan Meier plots for OS stratified based on different modes of inheritance for rs1799930 showed different trends. For instance, despite being statistically insignificant in the codominant mode stratification, patients with an AA genotype had a higher probability of survival after around 3 years, followed by those with GG and lastly patients with a heterozygous genotype of GA (median OS–AA: 33.7 months, GG: 30.8 months, GA: 9.1 months, P = 0.334 (**Fig 1**). Since there where only one *IDH* mutation (*IDH2* rs121913502), survival analysis was not feasible.

### TCGA mutational analysis

**Fig 2:**
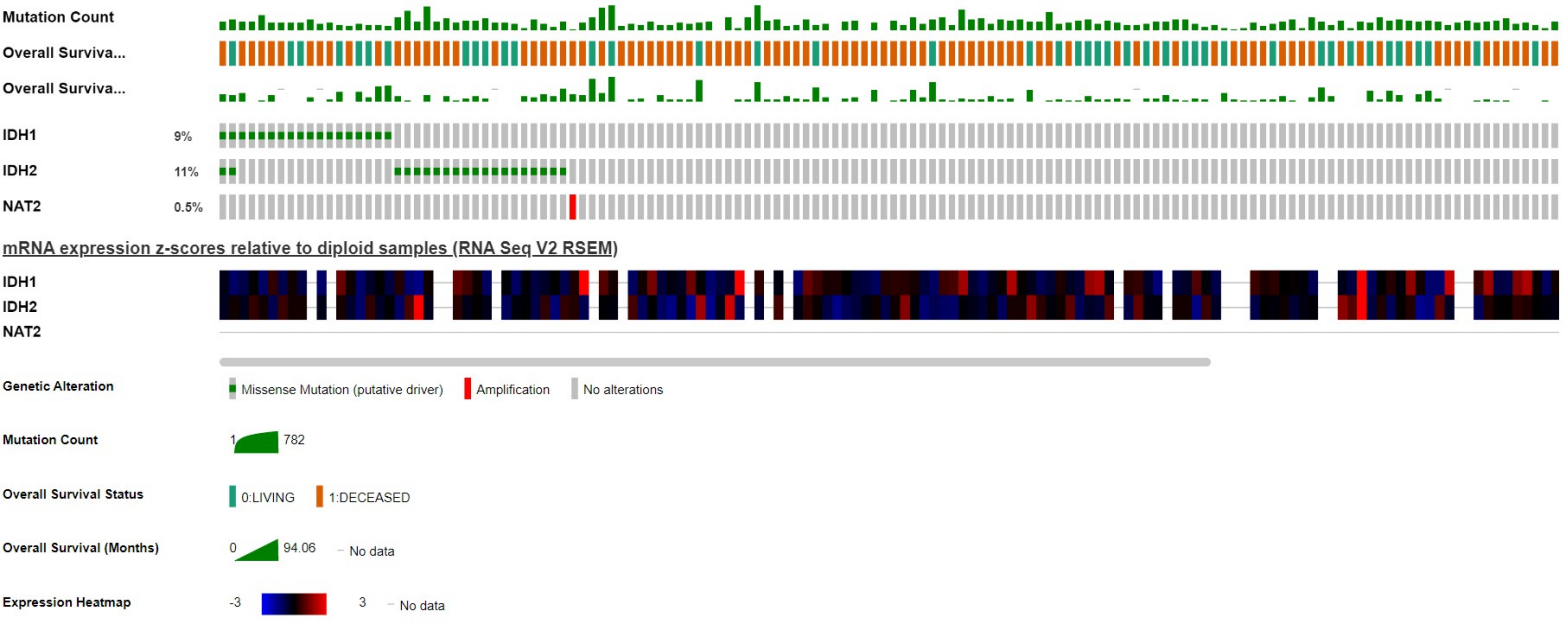
Oncoprint graph demonstrating the distribution of included genes mutations in the TCGA-AML cohort with clinical annotation for mutation count and overall survival.

**(Fig 2)** Illustrates an Oncoprint displaying the distribution of included genes’ mutations across the TCGA AML cohort coupled with an RNAseq heat-map showing corresponding expression. Interestingly, *IDH* mutations were reported in 20% of patients, whereas *NAT2* was only mutated in 1 patient which had an amplification mutation.

**Fig 3:**
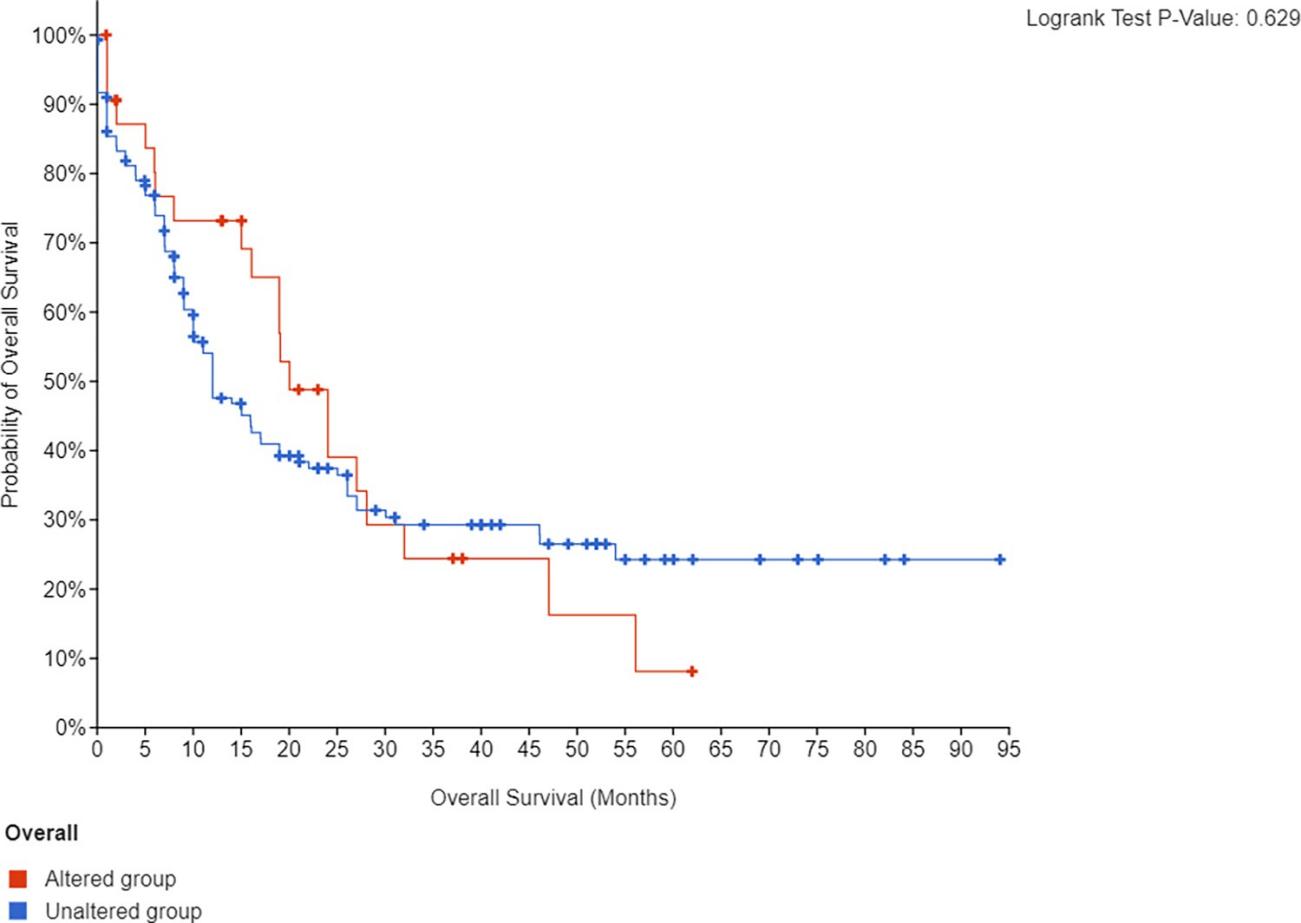
Kaplan Meier plots demonstrating the probability of overall survival of the TCGA-AML cohort (n = 200) based on the mutational status of *IDH1/2*.

As for *IDH* mutated patients there was no significant difference in OS regardless of the *IDH* type (**Fig 3**).

**Fig 4:**
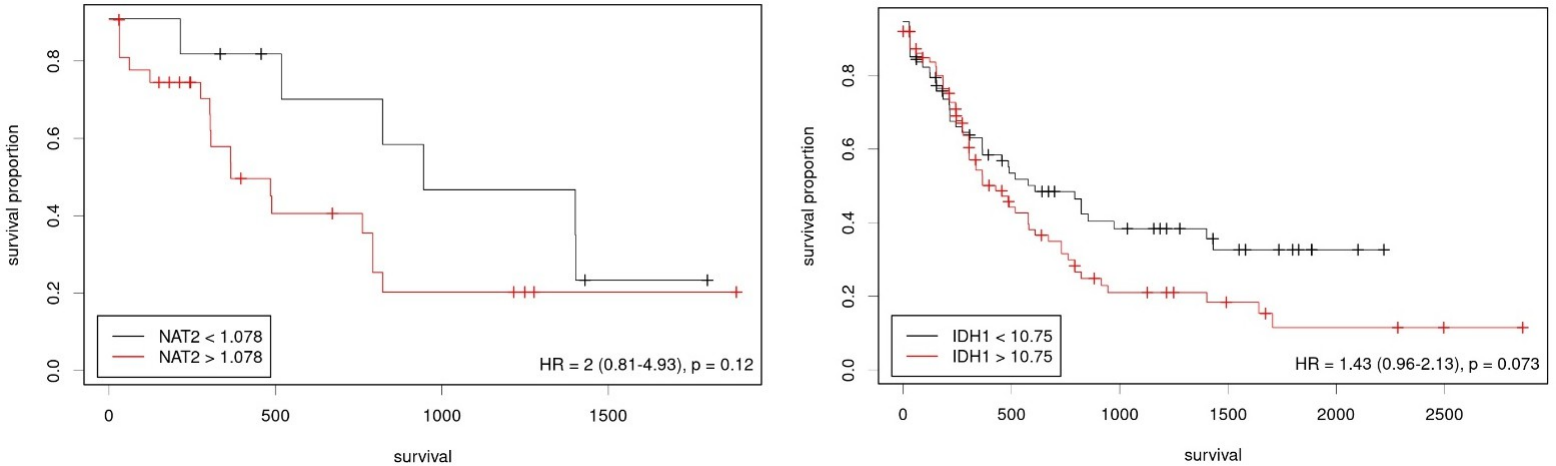
Kaplan Meier plots demonstrating the probability of survival with patients from the TCGA-AML cohort stratified based on the expression of included genes. High and low expression groups were stratified using a cut-off point calculated using the X-Tile program, (a) *NAT2* and (b) *IDH1*.

Included TCGA AML patients were stratified into high and low expression groups based on a cut-off point determined using the X-Tile software. Both *NAT2* and *IDH1* stratification did not result in a statistically significant difference although numerically favoring the lower expression group (**Fig 4**).

## Discussion

The impact of various SNPs has been widely studied in AML which in return has contributed to a deeper understanding of underlying mechanisms for leukemogenesis, treatment response as well as toxicity leading to improved prognosis and more adequate treatment strategies. Genetic variations are important to consider within the context of interpopulation variations, and our study serves to be the first from the region to investigate our SNPs of interest in AML patients. Among the five SNPs (*NAT2* (rs1799930 and rs1799931), *IDH1* (rs121913500), and *IDH2* (rs121913502 and rs1057519736)) that we analyzed to study the relationship between single nucleotide polymorphisms (SNPs) and the risk and overall survival of AML in Jordanian patients we found that *NAT2* rs1799930 is the only SNP to show a statistically significant difference in genotype frequency between cases and controls (p = 0.023), while other SNPs did not correlate with the risk and survival of AML in our population.

*NAT2* is a gene located on chromosome 8 that functions as an acetylator against different carcinogens with heterocyclic amines and amines with a carbon-only aromatic ring. Thus, *NAT2* might carry a protective role against cancer development. There are *NAT2* SNPs that affect the phenotype significantly, fast acetylators including rs1041983 and rs1799929 and slow acetylators including rs1799930 and rs1799931. We only investigated the role of the latter in the survival and risk of having AML. Kaplan Meier plots for OS stratified based on different modes of inheritance for rs1799930 showed different trends. For instance, despite being statistically insignificant in the codominant mode stratification, patients with an AA genotype had a higher probability of survival after around 3 years, followed by those with GG and lastly patients with a heterozygous genotype of GA (median OS–AA: 33.7 months, GG: 30.8 months, GA: 9.1 months, P = 0.334. In our study, rs1799930 *NAT2* was associated with a higher odd of having AML while rs1799931 did not show increased odds of having AML. A meta-analysis by Tian et al. showed that rs1799930 was a potential risk factor for having AML while rs1799931 was, surprisingly, a protective factor against different cancers [29]. In addition, these results were confirmed in acute leukemia by another meta-analysis by Zhu et al. in rs1799931 [19]. However, Jiang et al. found different results of no increased risk of having AML for both rs1799930 and rs1799931 [20]. To understand *NAT2*’s role in AML, we further studied the multi-omics data of the TCGA-LAML cohort. *NAT2* RNA-seq. expression was not correlated with better survival. *NAT2* mutation was rare as only one patient had a *NAT2* mutation. This might be due to the different ethnic groups between our Arab patients and the American patients in the TCGA cohort. On this note, Zhu et al. noted a different risk of acute leukemia in rs1799931 but not rs1799930 patients in studies that included mixed ethnic groups [19]. In addition, Caucasian rs1801280 patients were found to have the highest risk of acute leukemia. However, the results were limited by the heterogeneity of the included case-control studies.

Both *IDH1* and *IDH2* are among the most common mutations in AML. These mutations have been found to drive the formation of 2-hydroxyglutarate. High 2-hydroxyglutarate lead to a hypermethylated status, aberrant gene splicing, and impairment of hematopoietic differentiation [30]. Thus, Ivosidenib (*IDH1* inhibitor) and enasidenib (*IDH2* inhibitor) were developed and FDA-approved for R/R AML [22]. Our ability to further investigate the prognostic value of *IDH* mutations and to appropriately study the correlation between *IDH* mutations and overall survival in AML patients was jeopardized by the fact that we have limited sample size (only one patient with *IDH2* rs121913502 mutation) and failing to control confounding factors such as smoking. On the other hand, the incidence of the *IDH* mutations in the TCGA-LAML cohort was 20%. Still, the overall survival did not significantly differ based on *IDH* mutations. The reported prognostic role of *IDH1/2* mutations is still unclear. In a meta-analysis by Wang and colleagues [24], *IDH* mutations were not associated with OS (Hazard ratio [HR]: 1.05, 95% CI 0.89–1.23). However, when the data was stratified based on *IDH1* and *IDH2*, *IDH1* was correlated with worse OS (HR: 1.17, 95% CI 1.05–1.31) while *IDH2* was associated with better OS (HR: 0.78, 95% CI 0.66–0.93).

There were several limitations in our study. First, the sample size among the cases was small (n = 30). Second, other SNPs for the investigated genes were not assessed. Third, this study only covered the north Jordanian population. Fourth, patients’ exposure to possible carcinogens like smoking was not fully assessed. Finally, our survival analysis should be interpreted with caution as the retrospective design and small sample size might reflect inaccurate results.

## Conclusion

Our study indicates that rs1799930 *NAT2* is significantly present in AML patients while *IDH* mutations did not correlate with the risk and survival of AML in the Jordanian population. These results were also similar in the TCGA-LAML cohorts with the notable exception of the rare *NAT2* mutation. A larger cohort study is needed to further investigate our results.

## Supporting information

S1 Dataset (XLSX)

## References

[1] De Kouchkovsky I, Abdul-Hay M. “Acute myeloid leukemia: a comprehensive review and 2016 update.” *Blood Cancer J*. 2016;6(7). doi: 10.1038/bcj.2016.50 27367478 PMC5030376

[2] No Title. https://gco.iarc.fr/today/data/factsheets/populations/400-jordan-fact-sheets.pdf

[3] Infante MS, Piris MÁ, Hernández-Rivas JÁ. Molecular alterations in acute myeloid leukemia and their clinical and therapeutical implications. *Med Clin (Barc)*. 2018;151(9):362–367. doi: 10.1016/J.MEDCLI.2018.05.002 29895422

[4] Desai P, Mencia-Trinchant N, Savenkov O, et al. Somatic mutations precede acute myeloid leukemia years before diagnosis. *Nat Med*. 2018;24(7):1015–1023. doi: 10.1038/s41591-018-0081-z 29988143 PMC6849383

[5] Ouerhani S, Nefzi MA, Menif S, et al. Influence of genetic polymorphisms of xenobiotic metabolizing enzymes on the risk of developing leukemia in a Tunisian population. *Bull Cancer*. 2011;98(12). doi: 10.1684/BDC.2011.1502 22146408

[6] Puty TC, Sarraf JS, Do Carmo Almeida TC, et al. Evaluation of the impact of single-nucleotide polymorphisms on treatment response, survival and toxicity with cytarabine and anthracyclines in patients with acute myeloid leukaemia: A systematic review protocol. *Syst Rev*. 2019;8(1):1–8. doi: 10.1186/S13643-019-1011-Y/TABLES/1 31053175 PMC6499963

[7] Zou Y, Dong S, Xu S, Gong Q, Chen J. Genetic polymorphisms of NAT2 and risk of acute myeloid leukemia: A case-control study. *Medicine (Baltimore)*. 2017;96(42). doi: 10.1097/MD.0000000000007499 29049179 PMC5662345

[8] Alberg AJ, Daudt A, Huang HY, et al. N-acetyltransferase 2 (NAT2) genotypes, cigarette smoking, and the risk of breast cancer. *Cancer Detect Prev*. 2004;28(3):187–193. doi: 10.1016/j.cdp.2004.04.001 15225898

[9] Gra OA, Glotov AS, Kozhekbayeva ZM, Makarova O V., Nasedkina T V. Genetic polymorphism of GST, NAT2, and MTRR and susceptibility to childhood acute leukemia. *Mol* Biol 2008 422. 2008;42(2):187–197. doi: 10.1134/S0026893308020039 18610829

[10] Wichukchinda N, Pakdee J, Kunhapan P, et al. Haplotype-specific PCR for NAT2 diplotyping. *Hum Genome Var 2020 71*. 2020;7(1):1–6. doi: 10.1038/s41439-020-0101-7 32411379 PMC7214404

[11] Baranska M, Trzcinski R, Dziki A, Rychlik-Sych M, Dudarewicz M, Skretkowicz J. The role of N-acetyltransferase 2 polymorphism in the etiopathogenesis of inflammatory bowel disease. *Dig Dis Sci*. 2011;56(7):2073–2080. doi: 10.1007/s10620-010-1527-4 21321790 PMC3112481

[12] Ungcharoen U, Sriplung H, Mahasirimongkol S, et al. The Influence of NAT2 Genotypes on Isoniazid Plasma Concentration of Pulmonary Tuberculosis Patients in Southern Thailand. *Tuberc Respir Dis (Seoul)*. 2020;83(Supple 1):S55–S62. doi: 10.4046/trd.2020.0068 33138342 PMC7837378

[13] Tan EK, Khajavi M, Thornby JI, Nagamitsu S, Jankovic J, Ashizawa T. Variability and validity of polymorphism association studies in Parkinson’s disease. *Neurology*. 2000;55(4):533–538. doi: 10.1212/wnl.55.4.533 10953187

[14] Zhu Z, Zhang J, Jiang W, Zhang X, Li Y, Xu X. Risks on N-acetyltransferase 2 and bladder cancer: a meta-analysis. *Onco Targets Ther*. 2015;8:3715. doi: 10.2147/OTT.S82927 26715854 PMC4685932

[15] Zhu K, Xu A, Xia W, et al. Association Between NAT2 Polymorphism and Lung Cancer Risk: A Systematic Review and Meta-Analysis. *Front Oncol*. 2021;11:102. doi: 10.3389/fonc.2021.567762 33777732 PMC7991837

[16] Agundez JAG, Lozano L, Ladero JM, et al. N-Acetyltransferase 2 (NAT2) Genotype and Colorectal Carcinoma: Risk Variability According to Tumour Site? *http://dx.doi.org/10.1080/003655200451225*. 2009;35(10):1087–1091. 10.1080/003655200451225 11099063

[17] Boccia S, Sayed-Tabatabaei FA, Persiani R, et al. Polymorphisms in metabolic genes, their combination and interaction with tobacco smoke and alcohol consumption and risk of gastric cancer: a case-control study in an Italian population. *BMC* cancer [electronic Resour. 2007;7:206–206. doi: 10.1186/1471-2407-7-206 17996038 PMC2194718

[18] Zanrosso CW, Emerenciano M, Faro A, De Aguiar Gonçalves BA, Mansur MB, Pombo-De-Oliveira MS. Genetic variability in N-acetyltransferase 2 gene determines susceptibility to childhood lymphoid or myeloid leukemia in Brazil. *Leuk Lymphoma*. 2012;53(2):323–327. doi: 10.3109/10428194.2011.619605 21888617

[19] Zhu X, Liu Y, Chen G, et al. Association between NAT2 polymorphisms and acute leukemia risk: A meta-analysis. *Medicine (Baltimore)*. 2019;98(12). doi: 10.1097/MD.0000000000014942 30896661 PMC6709067

[20] Jiang WH, Wang XT, Zheng LD, Yan QQ, Chen LL. Relationship between NAT2 polymorphisms and onset risk of acute leukemia: A systematic review and meta-analysis. *Eur Rev Med Pharmacol Sci*. 2019;23(21):9259–9266. doi: 10.26355/eurrev_201911_19418 31773677

[21] Weller M, Wick W, Aldape K, et al. Glioma. *Nat Rev Dis Prim*. 2015;1. doi: 10.1038/NRDP.2015.17 27188790

[22] Issa GC, DiNardo CD. Acute myeloid leukemia with IDH1 and IDH2 mutations: 2021 treatment algorithm. *Blood Cancer J 2021 116*. 2021;11(6):1–7. doi: 10.1038/s41408-021-00497-1 34083508 PMC8175383

[23] Dang L, White DW, Gross S, et al. Cancer-associated IDH1 mutations produce 2-hydroxyglutarate. *Nat 2009 4627274*. 2009;462(7274):739–744. doi: 10.1038/nature08617 19935646 PMC2818760

[24] Xu Q, Li Y, Lv N, et al. Correlation Between Isocitrate Dehydrogenase Gene Aberrations and Prognosis of Patients with Acute Myeloid Leukemia: A Systematic Review and Meta-Analysis. *Clin Cancer Res*. 2017;23(15):4511–4522. doi: 10.1158/1078-0432.CCR-16-2628 28246275

[25] Genomic and Epigenomic Landscapes of Adult De Novo Acute Myeloid Leukemia. *N Engl J Med*. 2013;368(22):2059–2074. doi: 10.1056/NEJMOA1301689/SUPPL_FILE/NEJMOA1301689_DISCLOSURES.PDF 23634996 PMC3767041

[26] Cerami E, Gao J, Dogrusoz U, et al. The cBio cancer genomics portal: an open platform for exploring multidimensional cancer genomics data. *Cancer Discov*. 2012;2(5):401–404. doi: 10.1158/2159-8290.CD-12-0095 22588877 PMC3956037

[27] Goldman MJ, Craft B, Hastie M, et al. Visualizing and interpreting cancer genomics data via the Xena platform. *Nat Biotechnol*. 2020;38(6):675–678. doi: 10.1038/s41587-020-0546-8 32444850 PMC7386072

[28] Camp RL, Dolled-Filhart M, Rimm DL. X-tile: a new bio-informatics tool for biomarker assessment and outcome-based cut-point optimization. *Clin Cancer Res*. 2004;10(21):7252–7259. doi: 10.1158/1078-0432.CCR-04-0713 15534099

[29] Tian FS, Shen L, Ren YW, Zhang Y, Yin ZH, Zhou B Sen. N-acetyltransferase 2 gene polymorphisms are associated with susceptibility to cancer: a meta-analysis. *Asian Pac J Cancer Prev*. 2014;15(14):5621–5626. doi: 10.7314/APJCP.2014.15.14.5621 25081676

[30] Montalban-Bravo G, DiNardo CD. The role of IDH mutations in acute myeloid leukemia. *https://doi.org/10.2217/fon-2017-0523*. 2018;14(10):979–993. 10.2217/fon-2017-0523 29543066

